# Identification of dysregulated long non-coding RNAs/microRNAs/mRNAs in TNM I stage lung adenocarcinoma

**DOI:** 10.18632/oncotarget.18512

**Published:** 2017-06-16

**Authors:** Ziqiang Tian, Shiwang Wen, Yuefeng Zhang, Xinqiang Shi, Yonggang Zhu, Yanzhao Xu, Huilai Lv, Guiying Wang

**Affiliations:** ^1^ Department of Thoracic Surgery, The Fourth Hospital of Hebei Medical University, Shijiazhuang, China; ^2^ The Second Department of Surgery, The Fourth Hospital of Hebei Medical University, Shijiazhuang, China

**Keywords:** TNM I stage, lung adenocarcinoma, long non-coding RNA, microRNA, RNA-sequencing

## Abstract

Lung adenocarcinoma (LUAD) is the primary subtype in lung cancer, which is the leading cause of cancer-related death worldwide. This study aimed to investigate the aberrant expression profiling of long non-coding RNA (lncRNA) in TNM I stage (stage I) LUAD. The lncRNA/mRNA/miRNA expression profiling of stage I LUAD and adjacent non-tumor tissues from 4 patients were measured by RNA-sequencing. Total of 175 differentially expressed lncRNAs (DELs), 1321 differentially expressed mRNAs (DEMs) and 94 differentially expressed microRNAs (DEMIs) were identified in stage I LUAD. DEMI-DEM regulatory network consisted of 544 nodes and 1123 edge; miR-200 family members had high connectivity with DEMs. In DEL-DEM co-expression network, CDKN2B-AS1, FENDRR and LINC00312 had the high connectivity with DEMs, which co-expressed with 105, 63 and 61 DEMs, respectively. DEL-DEMI-DEM network depicted the links among DELs, DEMI and DEMs. Identified DEMs were significantly enriched in cell adhesion molecules, focal adhesion and tight junction of Kyoto Encyclopedia of Genes and Genomes (KEGG) pathways; and enriched in cell adhesion, angiogenesis and regulation of cell proliferation of Gene Ontology biological processes. Quantitative real-time polymerase chain reaction results were generally consistent with our bioinformatics analyses. LINC00312 and FENDRR had diagnostic value for LUAD patients in The Cancer Genome Atlas database. Our study might lay the foundation for illumination of pathogenesis of LUAD and identification of potential therapeutic targets and novel diagnosis biomarkers for LUAD patients.

## INTRODUCTION

Lung adenocarcinoma (LUAD), the primary histological subtype of non-small cell lung cancer (NSCLC), is the leading cause of cancer mortality both in female and male around the world [1].

Resection surgery is recognized as the radical treatment for patients with LUAD, However, patients frequently lose opportunity for radical resection on account of most of patients are diagnosed with advanced stage LUAD in hospitalization [2]. In addition, chemotherapy and radiotherapy are the standard first- and second-line treatments for patients with LUAD, but the curative effect remains undesirable [3]. Although epidermal growth factor receptor tyrosine kinase inhibitors (EGFR-TKIs), such as gefitinib and erlotinib, greatly improve the life quality of patients, but EGFR-TKIs cannot significantly prolong overall survival and results in inevitable gefitinib-resistance [4]. In spite of the aforementioned advances in clinical therapy, the prognosis 5- year overall survival of patients with LUAD is dissatisfying. Although it is known that early diagnosis and treatment for patients with LUAD will be beneficial to prolong overall survival of patients, but the current diagnostic biomarker for early stage LUAD is not feasible in clinical practice on account of unsatisfactory sensitivity and specificity.

Long non-coding RNAs (lncRNAs) are those transcripts with length more than 200 nt and not translated into proteins. Mounting evidences indicate that aberrant expression of lncRNAs is involved in modulating the transcription and translation of protein-coding genes at the transcriptional level, post-transcriptional level and epigenetic level [5]. lncRNAs play essential roles in cell proliferation and metastasis of various cancer types. Ectopic expression of AFAP-AS1 promotes cell proliferation and inhibits cell apoptosis in esophageal squamous cell carcinoma (ESCC), which is significantly correlated with advanced TNM stage and larger tumor size [6]. Over-expression of lncRNA MALAT1 predicts poor recurrence-free survival in tamoxifen treated ER-positive breast cancer patients [7]. In gallbladder, high expression level of MALAT1 is correlated with larger tumor size, lymphatic metastasis and shorter overall survival; silencing of MALAT1 inhibits cell proliferation, cell invasion and increases cell apoptosis [8]. In currently, a few of articles demonstrate the lncRNA expression profiling in LUAD tissues, but the global lncRNA expression profiling in TNM I stage LUAD (stage I LUAD) is not uncovered.

In our work, lncRNA, miRNA and mRNA expression profiling of patients with early stage LUAD were obtained through RNA-sequencing, abnormally expressed lncRNAs, miRNAs and mRNAs were identified, and lncRNA/mRNA co-expression network were constructed. Our study might provide new insights into investigation of tumorigenesis mechanism in LUAD and discover of candidate diagnostic biomarkers and therapeutic targets for LUAD.

## RESULTS

### Differentially expressed lncRNAs and mRNAs in TNM I stage LUAD

Respective 15710 lncRNAs and 22528 mRNAs were mapped with the human reference genome Ensemble GRCh38 v 84. Total of 175 differentially expressed lncRNA (DELs, 63 up- and 112 down-regulated) and 1321 differentially expressed mRNA (DEMs, 587 up- and 734 down-regulated) were identified in TNM I stage LUAD tissues compared to paired non-tumor tissues according to the threshold of *P*<0.05 and |log_2_Fold change|≥1. 175 DELs and 1321 DEMs were widely distributed in all autosomes and chromosomes X (Supplementary Figure 1). The top 15 up- and down-regulated DELs/DEMs were shown in Tables 1 and 2. LOC80078 and LOC101930114 were the most significantly up- and down-regulated DELs; EEF1A2 and ANKRD1 were the most significantly up- and down-regulated DEMs in TNM I stage LUAD tissues compared to paired non-tumor tissues (Tables 1 and 2).

**Table 1:** Top 15 up- and down-regulated DELs in stage I LUAD

lncRNAs	Chromosome	Position	log_2_FC	*P*-value
**Up-regulation**				
LOC80078	chr6	79307668-79313384	8.959	0.02875
LOC105371855	chr17	62701313-62808381	6.49313	0.0358
HNF1A-AS1	chr12	120969837-120972292	6.30907	0.0176
LINC00858	chr10	84279979-84294659	5.68535	0.0238
LOC101926969	chr2	91571574-91580863	4.86016	0.0358
LOC442497	chr7	379424-382879	4.42594	0.0328
LOC105374398	chr4	35981204-35985266	4.26679	0.02985
LOC105371082	chr16	11249045-11523533	3.95453	0.0459
RAB30-AS1	chr11	83072065-83073712	3.42599	0.0424
CDKN2B-AS1	chr9	21967138-22121097	3.11379	0.0315
LOC105370333	chr13	100086227-100088973	3.04753	0.04915
LOC105374994	chr6	26813826-26827989	3.03417	0.0422
LOC105375172	chr7	17374916-17558909	2.79503	0.00005
LINC01106	chr2	110375108-110384843	2.73542	0.0033
HAGLR	chr2	176173188-176190907	2.03736	0.00005
**Down-regulation**				
LOC101930114	chr1	209661358-209784545	-5.71679	0.0011
LOC102724660	chr7	128434062-128469400	-5.61929	0.04095
LOC105369761	chr12	49539040-49568225	-5.43482	0.0215
LOC100507487	chr4	128428015-128519398	-5.00157	0.04675
TUSC7	chr3	116709787-116717040	-4.69494	0.0344
LOC105378016	chr6	136557045-136793051	-4.6798	0.04305
NR2F2-AS1	chr15	96127359-96340263	-4.62638	0.03075
LOC101928926	chr19	19756370-19791747	-4.29708	0.0107
LOC105371720	chr17	30236905-30292166	-4.11397	0.03245
LOC105370818	chr15	51915876-51971801	-4.10768	0.00825
FAM95B1	chr9	40323570-40329220	-3.95732	0.0243
SALRNA2	chr15	70635248-70637314	-3.88695	0.0369
LOC101930496	chr17	83110232-83119423	-3.59853	0.03035
LOC284865	chr22	20198729-20204918	-3.58415	0.0288
LOC105370168	chr13	39208770-39227906	-3.57939	0.0424

DELs: differentially expressed long non-coding RNAs; LUAD: lung adenocarcinoma; FDR: false discovery rate; FC: fold change; chr: chromosome.

**Table 2:** Top 15 up- and down-regulated DEMs in stage I LUAD

mRNAs	Chromosome	Position	log_2_FC	*P*-value
**Up-regulation**				
EEF1A2	chr20	63488011-63499315	7.95929	0.0456
CEACAM5	chr19	41708610-41730433	6.26554	0.0002
CLDN2	chrX	106900062-106930861	5.83291	0.00005
HABP2	chr10	113550830-113664070	5.77961	0.01665
COL11A1	chr1	102876466-103108576	5.49743	0.008
SLC9A2	chr2	102619688-102711350	5.29808	0.0217
PTPRZ1	chr7	121873104-122062036	5.25139	0.00325
MMP13	chr11	102942991-102955734	5.02238	0.01595
LOC100129940	chr12	65758019-65966291	4.97897	0.0378
TOX3	chr16	52436424-52547802	4.77916	0.0247
ETV4	chr17	43527842-43546432	4.73691	0.0109
HS6ST2	chrX	132626009-132961395	4.63563	0.01275
MMP7	chr11	102520507-102530747	4.58386	0.00005
DKK1	chr10	52314280-52317657	4.30752	0.04615
TMPRSS4	chr11	118015771-118125505	4.26309	0.0133
**Down-regulation**				
ANKRD1	chr10	90912099-90921275	-7.62319	0.04905
TRIM58	chr1	247857198-247880138	-5.23987	0.0444
CST6	chr11	66011990-66013505	-4.39682	0.0237
SLC5A4	chr22	32218420-32353008	-4.2162	0.0051
SLC6A4	chr17	30194318-30235968	-4.11118	0.00025
MGAT5B	chr17	76850502-76950393	-3.92805	0.0085
TMEM100	chr17	55719626-55732121	-3.91121	0.00025
LOC105375355	chr7	76510101-76544090	-3.84847	0.03815
ADGRE2	chr19	14689786-14778541	-3.67241	0.00015
KIF19	chr17	74326211-74355820	-3.50716	0.0445
DTHD1	chr4	36281610-36345756	-3.34849	0.037
FCN3	chr1	27369109-27374825	-3.32501	0.0024
ZMYND10	chr3	50341105-50346028	-3.28484	0.0329
BTNL9	chr5	181040224-181061523	-3.03646	0.0001
VIPR1	chr3	42489298-42537573	-3.00424	0.00005

DEMs: differentially expressed mRNAs; LUAD: lung adenocarcinoma; FDR: false discovery rate; FC: fold change; chr: chromosome.

### Differentially expressed miRNA (DEMIs) in stage I LUAD

Total of 94 DEMIs including 87 up- and 7 down-regulated DEMIs were identified in TNM I stage LUAD tissues compared with paired non-tumor tissues based on the threshold of FDR<0.001 and |log_2_fold change|≥1. hsa-miR-194-5p, hsa-miR-135b-5p and hsa-miR-215-3p were significantly up-regulated in stage I LUAD tissues; hsa-miR-486-5p, hsa-miR-338-3p, hsa-miR-7641, hsa-miR-138-5p, hsa-miR-451a, hsa-miR-486-3p and hsa-miR-139-3p were significantly down-regulated in early stage LUAD tissues compared with adjacent non-tumor tissues (Supplementary Table 1).

### Construction of DEMI-DEM interaction network

**Figure 1:**
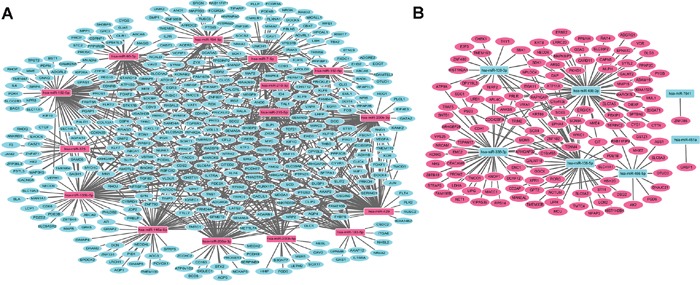
DEMI-DEM regulatory network in stage I lung adenocarcinoma **(A)** The network among up-regulated DEMIs and down-regulated DEMs. **(B)** The network among down-regulated DEMIs and up-regulated DEMs. The rectangle node and circular node indicated DEMIs and DEMs, respectively; the rose color and turquoise color represented up-regulation and down-regulation, respectively. DEMIs indicated differentially expressed microRNAs; DEMs were differentially expressed protein-coding mRNAs.

The target-genes of top 15 up- and down-regulated DEMIs in stage I LUAD were predicted though miRWalk database. Those predicted target-genes were overlapped with 1321 DEMs, and DEMI-DEM interaction pairs in a negative regulation manner were our concern due to miRNA commonly negatively regulates the expression of mRNA. DEMI-DEM regulatory network were visualized by Cytoscape software. As Figure 1A shown, up-regulated DEMIs/down-regulated DEMs regulatory network composed of 395 nodes and 942 edges, which was involved in 15 up-regulated DEMIs and 384 DEMs; hsa-miR-182-5p, hsa-miR-200b-3p and hsa-miR-429 had the highest connectivity with down-regulated DEMs, which interacted with 99, 83 and 82 DEMs, respectively. As Figure 1B shown, down-regulated DEMIs/up-regulated DEMs regulatory network composed of 139 nodes and 181 edges, which was involved in 7 up-regulated DEMIs and 132 DEMs; hsa-miR-486-3p, hsa-miR-138-5p and hsa-miR-338-3p had the highest connectivity with up-regulated DEMs, which interacted with 61, 45 and 41 down-regulated DEMs, respectively.

### The nearby DEMs of DELs in reference genome

**Figure 2:**
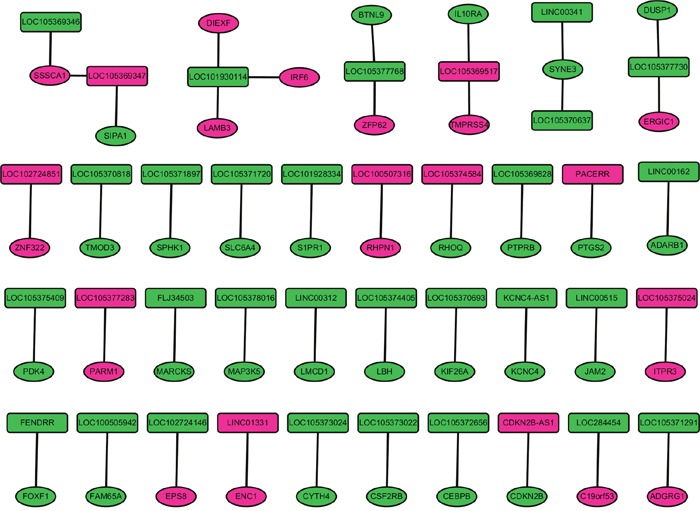
The nearby DEMs of DELs in stage I lung adenocarcinoma The rectangle and circular nodes indicated DELs and DEMs, respectively; rosecolor and green color indicated up- and down-regulation, respectively. DELs indicated differentially expressed long non-coding RNAs; DEMs were differentially expressed protein-coding mRNAs.

The nearby protein-coding genes of 175 DELs with distance<100kb were identified in GRCH38 reference genome. Total 190 nearby genes of 195 DELs were identified. Those 190 genes were overlapped with 1321 DEMs in stage I LUAD, and 42 nearby DEMs of 38 DELs were available, as Figure 2 shown. IRF6, DIEXF and LAMB3 were the nearby DEMs of LOC101930114.

### DEL/DEM co-expression network construction

In order to investigate the potential functions of DELs, DEL/DEM co-expression network was constructed. PCC of each DEL-DEM co-expression pair among 175 DELs and 1321 DEMs was calculated based on the expression level of DELs/DEMs.

**Figure 3:**
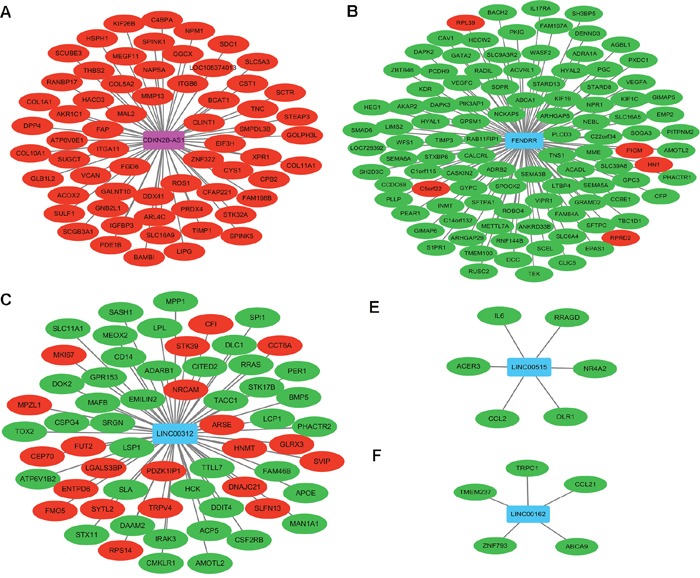
Co-expression network among DELs and DEMs in stage I lung adenocarcinoma **(A)** Sub-network of CDKN2B-AS1; **(B)** sub-network of FENDRR; **(C)** sub-network of LINC00312; **(D)** sub-network of LINC00515; **(E)** sub-network of LINC00162. Rectangle nodes and circular nodes represented DELs and DEMs, respectively. Rose color and red color indicated up-regulation; blue color and green color indicated down-regulation.

DEL-DEM co-expression pairs with |PCC>0.9| was subjected to network construction. In Figure 3 shown, CDKN2B-AS1, FENDRR, LINC00312, LINC00515 and LINC00162 co-expressed with 105, 63, 61, 6 and 5 DEMs, respectively.

### DEL-DEMI-DEM network

**Figure 4:**
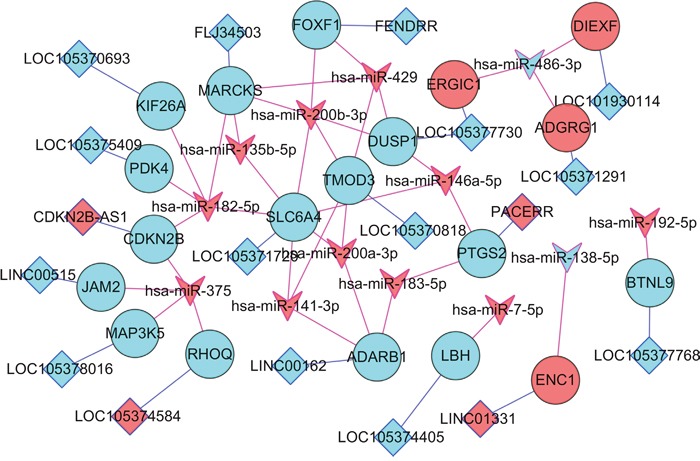
DEL-DEMI-DEM network Diamond nodes and circular nodes indicated DELs and DEMs, respectively. V nodes represented DEMIs. Blue line indicated the nearby link between DEL and DEM; red line indicated the negative correlation between DEMI and DEM. Red color and turquoise color was up- and down-regulation. DELs indicated differentially expressed long non-coding RNAs; DEMs were differentially expressed protein-coding mRNAs; DEMIs indicated differentially expressed microRNAs.

In our study, DEL-DEMI-DEM network were constructed. As Figure 4 shown, DEMI-DEM interaction pairs and the nearby DEMs of DELs were depicted in the network. For instance, in Figure 4, FOXF1 was the nearby DEM of lncRNA FENDRR and were negatively regulated by hsa-miR-200b-3p and hsa-miR-439. CDKN2B was the nearby DEM of lncRNA CDKN2B-AS1 and was negatively targeted by hsa-miR-182-5p and hsa-miR-375.

### GO and KEGG pathway enrichment

1321 DEMs were significantly enriched in pathways in cancer (Kegg:05200), cell adhesion molecules(Kegg:04514), focal adhesion (Kegg:04510), cytokine-cytokine receptor interaction (Kegg:04060), ECM-receptor interaction (Kegg:04512) and tight junction (Kegg:04530) (Table 3). Furthermore, those DEMs were obviously enriched in signal transduction, cell adhesion, angiogenesis, regulation of cell proliferation, regulation of cell migration, regulation of cell apoptosis and response to hypoxia of Gene Ontology (GO) biological process (Table 3).

**Table 3:** GO biological process and KEGG signaling pathway enrichment of DEMs in stage I LUAD

Items	Details	Support genes	FDR
**GO-biological process enrichment (top 15)**			
GO:0007165	Signal transduction	135	2.90E-29
GO:0007155	Cell adhesion	73	2.74E-18
GO:0001525	Angiogenesis	35	6.29E-15
GO:0007275	Multicellular organismal development	89	1.89E-13
GO:0008284	Positive regulation of cell proliferation	47	2.08E-11
GO:0007596	Blood coagulation	53	4.95E-11
GO:0030335	Positive regulation of cell migration	24	1.37E-10
GO:0001666	Response to hypoxia	30	4.56E-10
GO:0030154	Cell differentiation	55	4.89E-10
GO:0007275	Multicellular organismal development	44	6.01E-10
GO:0042493	Response to drug	38	5.85E-09
GO:0043066	Negative regulation of apoptotic process	35	1.76E-08
GO:0001501	Skeletal system development	24	1.81E-08
GO:0008285	Negative regulation of cell proliferation	39	5.06E-08
GO:0009612	Response to mechanical stimulus	14	5.52E-08
**KEGG pathway enrichment (top 15)**			
Kegg:05200	Pathways in cancer	37	2.92E-07
Kegg:04514	Cell adhesion molecules (CAMs)	20	2.02E-06
Kegg:04510	Focal adhesion	26	2.30E-06
Kegg:04060	Cytokine-cytokine receptor interaction	30	2.64E-06
Kegg:04512	ECM-receptor interaction	16	3.19E-06
Kegg:04360	Axon guidance	19	8.68E-06
Kegg:04610	Complement and coagulation cascades	13	1.94E-05
Kegg:04144	Endocytosis	23	2.17E-05
Kegg:04380	Osteoclast differentiation	18	2.25E-05
Kegg:05144	Malaria	11	2.52E-05
Kegg:04530	Tight junction	18	2.76E-05
Kegg:04666	Fc gamma R-mediated phagocytosis	15	2.82E-05
Kegg:04350	TGF-beta signaling pathway	14	3.09E-05
Kegg:05219	Bladder cancer	9	0.000171
Kegg:05140	Leishmaniasis	8	0.00027

GO: gene ontology; KEGG: Kyoto Encyclopedia of Genes and Genomes; FDR: false discovery rate; DEMs: differentially expressed mRNAs; LUAD: lung adenocarcinoma.

### qRT-PCR validation of the expression levels of dysregulatedDEMIs/DEMs in stage I LUAD

**Figure 5:**
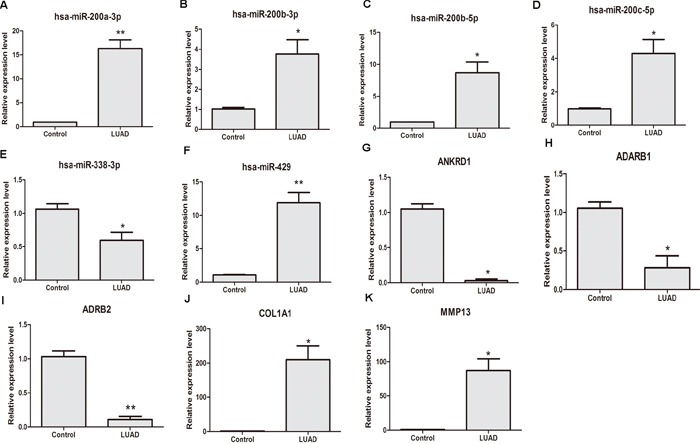
qRT-PCR validation of dysregulated DEMIs/DEMs in stage I LUAD compared with adjacent non-tumor tissues **(A)** hsa-miR-200a-3p; **(B)** hsa-miR-200b-3p; **(C)** hsa-miR-200b-5p; **(D)** hsa-miR-200c-5p; **(E)** hsa-miR-338-5p; **(F)** hsa-miR-428; **(G)** ADARB1; **(H)** ADRB2; **(I)** ANKRD1; **(J)** COL1A1; **(K)** MMP13. LUAD indicated stage I lung adenocarcinoma tissues; control indicated paired adjacent non-tumor tissues. * represented *P*<0.05 and ** represented *P* <0.01.

qRT-PCR was subjected to validate the expression levels of dysregulated DEL/DEMI/DEM in 6 stage I LUAD tissues and 6 adjacent non-tumor tissues. 3 DELs, 5 DEMIs and 6 DEMs were used to apply for qRT-PCR verification. As Figure 5A-5E shown, hsa-miR-200a-3p (*P*<0.01), hsa-miR-200b-3p (*P*<0.05), hsa-miR-200b-5p (*P*<0.05), hsa-miR-200c-5p (*P*<0.05) and hsa-miR-429 (*P*<0.01) were obviously up-regulated in stage I LUAD tissues and hsa-miR-338-3p (*P*<0.05) was obviously down-regulated in stage I LUAD tissues compared with adjacent non-tumor tissues. The expression levels of ADARB1 (*P*<0.01), ADRB2 (*P*<0.05) and ANKRD1 (*P*<0.05) were significantly down-regulated in stage I LUAD tissues; COL1A1 (*P*<0.05) and MMP13 (*P*<0.05) were significantly up-regulated in stage I LUAD tissues compared with adjacent non-tumor tissues (Figure 5G-5K).

### The expression levels of candidate DELs in LUAD were analyzed based on TCGA datasets and qRT-PCR experiments

**Figure 6:**
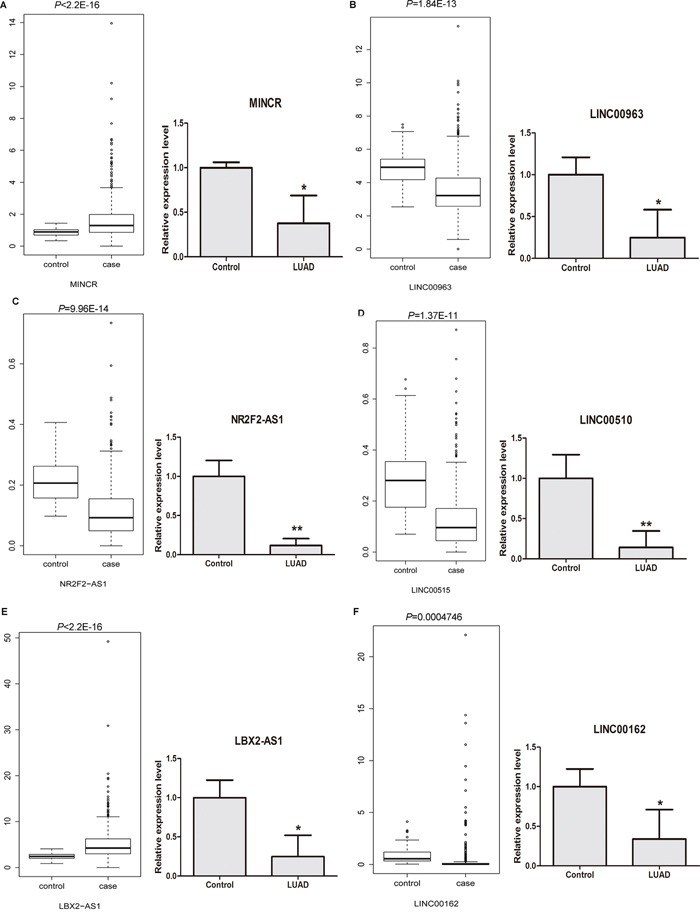
The cross validation of the expression levels of candidate DELs in LUAD tissues based on TCGA database and qRT-PCR experiments **(A)** MINCR; **(B)** LINC00963; **(C)** NR2F2-AS1; **(D)** LINC00515; **(E)** LBX2-AS1; **(F)** LINC00162. Case group and control group indicated LUAD tissues and adjacent non-tumor tissues, respectively. LUAD indicated lung adenocarcinoma tissues. * represented *P*<0.05 and ** represented *P* <0.01.

The lncRNA expression profiling of LUAD tissues (case group) and adjacent non-tumor tissues (control group) were retrieved from TCGA database. In addition, the expression of 12 candidate DELs in LUAD tissues was detected through qRT-PCR methods in 6 stage I LUAD tissues and 6 adjacent non-tumor tissues. As Figure 6A and 6E shown, both of lncRNA MINCR (Figure 6A) and LBX2-AS1 (Figure 6E) weresignificantlyup-regulated in LUAD tissues based on TCGA dataset, however, thosetwo lncRNAweresignificantly down-regulated in LUAD tissues based on qRT-PCR results. In Figure 6B, 6C, 6D and 6F, LINC00963 (Figure 6B), NR2F2-AS1 (Figure 6C), LINC00515 (Figure 6D), and LINC00162 (Figure 6F) were significantly down-regulated in LUAD tissues both in TCGA dataset and qRT-PCR experiment. As Supplementary Figure 2A-2D shown, LINC00312 (Supplementary Figure 2A), MGC27382 (Supplementary Figure 2B), LINC00472 (Supplementary Figure 2C) and FENDRR (Supplementary Figure 2D) were significantly down-regulated in LUAD tissues both inTCGA dataset and qRT-PCR experiment. CDKN2B-AS1 was significantly up-regulated in LUAD tissues based on TCGA dataset and it had the up-regulated tendency in LUAD tissues based on qRT-PCR (Supplementary Figure 2E). HNF1A-AS1 was significantly up-regulated in LUAD tissues based on TCGA dataset and had the down-regulated tendency in LUAD tissues based on qRT-PCR results (Supplementary Figure 2F).

Combined with the bioinformatics analyses of our RNA-sequencing, the expression 8 of 12 candidate lncRNAs including LINC00963, NR2F2-AS1, LINC00515, LINC00162, LINC00312, MGC27382, LINC00472 and FENDRR were significantly up-regulated in LUAD tissues based on our RNA-sequencing, TCGA dataset and qRT-PCR experiment. CDKN2B-AS1 was significantly up-regulated in LUAD tissues based on our RNA-sequencing and TCGA dataset, and it had the up-regulated tendency in LUAD tissues based on qRT-PCR results. MINCR and LBX2-AS1 were significantly up-regulated in LUAD tissues both in our RNA-sequencing and TCGA dataset and those two lncRNA was significantly down-regulated in LUAD tissues based on qRT-PCR. HNF1A-AS1 was significantly down-regulated in LUAD tissues both in our RNA-sequencing and qRT-PCR, and it is significantly up-regulated in LUAD tissues based on TCGA dataset.

In summary, the expression levels of 10 of 12 DELs in LUAD through qRT-PCR experiments were compatible with our RNA-sequencing; and the expression levels of 9 of 12 DELs in LUAD tissues through qRT-PCR experiments were compatible with the TCGA dataset. In general, the results of cross validation indicated that the expression patterns of candidate DELs in LUAD based on TCGA database and qRT-PCR was compatible with our RNA-sequencing and bioinformatics analyses.

### Receiver operating characteristic (ROC) curve analysis

**Figure 7:**
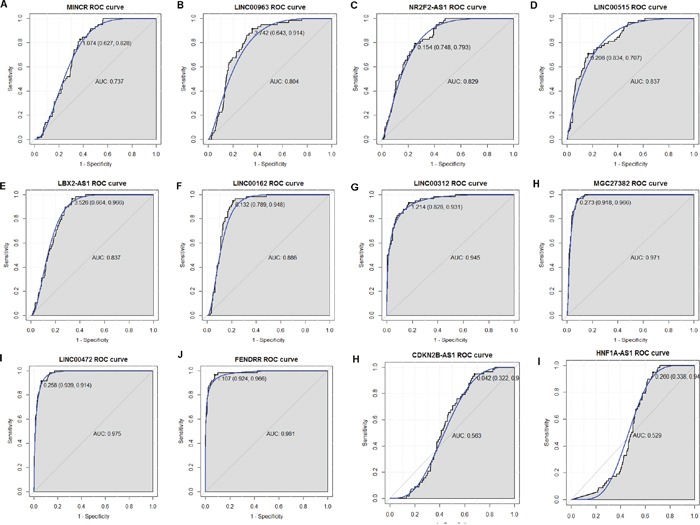
The discriminatory ability of DELs between LUAD tissues and adjacent non-tumor tissues was accessed with ROC curve **(A)** MINCR; **(B)** LINC00963; **(C)** NR2F2-AS1; **(D)** LINC00515; **(E)** LBX2-AS1; **(F)** LINC00162; **(G)** LINC00312; **(H)** MGC27382; **(I)** LINC00472; **(J)** FENDRR; **(K)** CDKN2B-AS1; **(L)** HNF1A-AS1.

In order to assess the discriminatory ability of the 12 candidate DELs among LUAD tissues and adjacent non-tumor tissues generated from TCGA database, ROC curve analyses were conducted and area under the curve (AUC) were calculated. As Figure 7A-7K shown, the AUC of 10 DELs was more than 0.7. The AUC of CDKN2B-AS1 and HNF1A-AS1 was respective 0.563 and 0.529, less than 0.7 (Figure 7L, 7K). LINC00312, MGC27382, LINC00472 and FENDRR had the largest AUC in those 12 DELs. For LUAD diagnosis, the sensitivity and specificity of LINC00312 was 93.1% and 82.8% (Figure 7G); the sensitivity and specificity of MGC27382 was 96.6% and 91.8% (Figure 7H); the sensitivity and specificity of LINC00472 was 91.4% and 93.9% (Figure 7I); the sensitivity and specificity of FENDRR was 96.6% and 92.4% (Figure 7J); respectively.

## DISCUSSION

In our study, all of the members of miR-200 family, such as miR-200a,-3p, miR-200a-5p, miR-200b-5p, miR-200b-3p, miR-200c-5p, miR-429, miR-141-5p and miR-200c-5p were obviously up-regulated (more than 2 fold) in stage I LUAD tissues compared to adjacent non-tumor tissue, which was accordance with the previous study [9]. The members of miR-200 family are the powerful regulator of epithelial-to-mesenchymal transition, which is essential in cell adhesion, cell invasion and cell metastasis in non-small cell lung cancer [9–12]. Increased miR-429 promotes cell proliferation and cell metastasis in NSCLC [13]. In addition to, serum levels of miR-141, miR-200b and miR-429 are potential biomarkers for early diagnosis in lung cancer [14, 15].

Recent evidence highlights lncRNA as vital regulators of cancer biology that contribute to cancer cell functions including cell proliferation, cell apoptosis, and metastasis. In our study, total of 75 DELs were identified in stage I LUAD.

LncRNA CDKN2B-AS1 (CDKN2B antisense RNA1, also named as ANRIL), a 3.8-kb-long RNA, transcribed from the short arm of human chromosome 9 on p21.3, was significantly up-regulated in stage I LUAD tissues compared to adjacent non-tumor tissues, which was consistent with the published article [16]. It is reported that CDKN2B-AS1 genetic polymorphisms are significantly associated with lung cancer susceptibility and response to platinum-based chemotherapy [17]. CDKN2B-AS1 is increased in lung cancer plasma samples, lung cancer tissues and lung cancer cell lines compared with those samples in healthy volunteers, adjacent non-tumor tissues and normal human bronchial epithelial cells [18]. Higher expression of CDKN2B-AS1 is significantly correlated with higher TNM stage, larger tumor size and shorter overall survival in patients with NSCLC [16]. CDKN2B encodes a cyclin-dependent kinase inhibitor and functions as the regulator of cell growth. CDKN2B is the nearby DEM of CDKN2B-AS1 and was obviously down-regulated in stage I LUAD tissues compared with adjacent non-tumor tissues. Copy number loss of CDKN2B predicts poor survival in patients with lung squamous cell carcinoma [19]. *CDKN2B* deficiency accelerates mutant KRAS lung tumorigenesis in mice mode, which leads to cell metastasis and cell proliferation [20]. In the DEL-DEM network, CDKN2B-AS1 co-expressed 63 up-regulated DEMs, such as MMP13, COL11A1, COL1A1, ITGA11, VACN, THBS2 and TIMP1, which were significantly enriched in cell adhesion molecules and focal adhesion pathways and enriched in cell adhesion, response to hypoxia, signal transduction and positive regulation of cell proliferation of GO biological process. In our previous study, THBS2, COL11A1 and VACN were also identified as up-regulated genes in NSCLC compared with adjacent non-tumor tissues through integrated analysis of microarray data [21]. Markedly over-expression of THBS2 and its co-expressed genes (such as VCAN, CLO11A1, FAP) predicts poor survival of patients with lung cancer; down-regulation of VCAN and THBS2 inhibits cell proliferation in NSCLC [22]. COL11A1 and COL1A1 belong to fibrillar collagen. COL11A1 is over-expressed in NSCLC with lymph node metastasis and recurrent NSCLC tissues, which promotes cell proliferation, cell migration, cell invasion and chemo-resistance [23]. The biological roles of COL1A1 in LUAD are unknown. Ectopic expression of MMP13 (matrix metallopeptidase 13) predicts poorer-5-year survival in patients with NSCLC [24].

In the DEL-DEM co-expression network, FENDRR (also named as FOXF1-AS1)had the high connectivity with DEMs, which co-expressed with 105 DEMs. Those DEMs were significantly enriched in pathways in cancer, focal adhesion and cytokine-cytokine receptor interaction pathways; and enriched in angiogenesis, regulation of cell proliferation, cell adhesion and signal transduction of GO biological process. FENDRR is identified as one of the most aberrantly expressed lncRNAs in human Xuanwei lung cancer through microarray analysis [25]. A recent published article demonstrates that over-expression of FENDRR inhibits cell migration, cell invasion and mediates stem-like properties by regulating epithelia-mesenchymal transition in NSCLC [26]. FOXF1 was the nearby DEM of FENDRR, and it is down-regulated in stage I LUAD tissues. Up-regulated FOXF1 has anti-malignant effects of mesenchymal stem cell fusion-induced reprogramming on lung cancer cells [27]. In the DEL-DEMI-DEM network, FOXF1 was negatively targeted by miR-200 family members of miR-429 and miR-200b-3p. ADRB2 was one of co-expressed genes with FENDRR and it was significantly down-regulated in stage I LUAD tissues. In our previous article, ADRB2 (encodes beta-2-adrenergic receptor, a member of the G protein-coupled receptor superfamily) is also down-regulated in NSCLC tissues compared with adjacent non-tumor tissues through integrated analysis of microarray data [21]. Genetic variants in ADRB2 confer the risk of chronic obstructive pulmonary disease and lung adenocarcinoma [28, 29]. The roles of ADRB2 in cell behaviors of LUAD including cell proliferation, cell invasiveness and cell metastasis are unclear.

LINC00162, LINC00515 and LINC00312 were significantly down-regulated in stage I LUAD tissues compared with adjacent non-tumor tissues. LINC00162 is P38 inhibited cutaneous squamous cell carcinoma associated lincRNA, also named as PICSAR and NLC1-C. Knockdown of LINC00162 suppresses cell proliferation and cell migration by inhibiting ERK1/2 activity and down-regulating the expression of DUSP6 in cutaneous squamous cell carcinoma [30]. Down-regulated LINC00162 promotes cell proliferation by repressing miR-320a and miR-383in testicular embrynonal carcinoma [31]. ADARB1 was the nearby DEM of LINC00162 and was down-regulated in stage I LUAD tissues. The expression level of ADARB1 in lung cancer cell lines is higher than that in normal human bronchial epithelial cells;higher mRNA expression level of ADARB1 predicts a better outcome in patients with lung adenocarcinoma. In addition to, ADARB1 was co-expressed with down-regulated LINC00312 (also named as NAG7). ADARB1 was targeted by miR-200 family members of miR-183-5p and miR-141, miR-200a-3p. Over-expression of LINC00312 inhibits cell migration and cell invasion in bladder cancer through down-regulation of miR-197-3p [32]. Lower expression of LINC00312 predicts larger tumor size and shorter overall survival in nasopharyngeal carcinoma [33]. Total of 61 dysregulated DEMs were co-expressed with LINC00312 and were enriched in tight junction, cell adhesion molecules and pathways in cancer pathways, and enriched in angiogenesis, cell adhesion and signal transduction of GO biological process. The expression of LINC00515 is decreased in cisplatin-resistant cells of high-grade serous ovarian cancer [34]. JAM2 was the nearby DEM of LINC00515. JAM2 encodes junctional adhesion molecule 2 and belongs to the immunoglobulin superfamily. Increased methylation of *JAM2* leads to down-regulation of JAM2, which is associated with LUAD pathogenesis [35]. JAM2 was significantly enriched in cell adhesion molecules and tight junction. Based on aforementioned information, LINC00162, LINC00515 and LINC00312 might play essential roles in tumorigenesis of LUAD and the biological functions of those dysregulated DELs in cell proliferation, invasiveness, metastasis and angiogenesis of LUAD are needed to be explored in the future work.

The ROC curve analyses indicated that LINC00312and FENDRRmight be potential biomarkers for LUAD diagnosis. In the further work, larger cohort of LUAD patients should be enrolled and the potential value of those lncRNAs in LUAD is needed to be further validated in clinical practice.

In conclusion, we identified the aberrantly expressed lnRNAs, miRNAs and mRNAs in stage I LUAD. Our study indicated that those dysregulated genes including miR-200 family, CDKN2B-AS1, FENDRR, LINC00162, LINC00515, LINC00312, ADARB1 and JAM2might synergistically contributes to tumorigenesis in stage I LUAD based on complex interactions between each other through KEGG pathways and GO biological processes including cell adhesion molecules, focal adhesion, tight junction, pathways in cancer, cell adhesion, angiogenesis and regulation of cell proliferation and regulation of cell apoptosis.

There are limitations in our study. Firstly, novel dysregulated lncRNAs were identified, such as LINC00162, LINC00515 and LINC00312, however, the biological roles of those genes involved in LUAD were not further investigated. Secondly, the diagnostic value of identified lncRNAs and miRNAs is needed to be translated into clinical practice through large cohort of LUAD patients. Our study might pave the way for illumination of pathogenesis of LUAD and discovery of potential therapeutic targets and novel diagnosis biomarkers for LUAD patients.

## MATERIALS AND METHODS

### Patients and samples

LUAD tumor tissues and paired adjacent non-tumor tissues for RNA-sequencing were obtained from patients who underwent surgery in the Fourth Hospital of Hebei Medical University. 4 patients with TNM I stage received none of chemo- or radiotherapy before resection surgery, were enrolled into our study (from Dec 29th, 2015 to Feb 18th, 2016). The pathological type and TNM stage of patients were explicitly diagnosed by histopathology examination. The basic information of patients including gender, age and TNM stage were recorded. The details were shown in Supplementary Table 2. This work was approved by the Ethics Committee of the Fourth Hospital of Hebei Medical University and informed written consent was obtained from all patients. The research complied with the principles of the Declaration of Helsinki.

### RNA-sequencing and data preprocessing

Total RNA of collected specimens was extracted by TRIzol reagent (Invitrogen, Carlsbad, CA, USA) for construction of cDNA libraries according to the manufacture instruction. Likewise, cDNA libraries of small RNA were constructed. Lastly, each library was conducted to IlluminaHiSeq 4000 sequencing.

The raw image data obtained from RNA-sequencing was translated into raw FASTQ sequence data. Adaptors of sequences were trimmed; nucleotides with a quality score <20 were trimmed from the end of the sequence; N base rate of raw reads more than 10% were discarded using Cutadapt 1.9.1. For microRNA (miRNA) sequencing, sequences with length less than 18ntor more than 32nt were abnegated. TopHat was used to align the clean reads of long non-coding RNAs (lncRNAs) and protein coding mRNAs (mRNAs) with the human reference genome, Ensemble GRCh38 v 84 (hg19) [36]. Moreover, the alignment of miRNAs with hg19 was implemented by Bowtie. Fragments per kilobase of exon per million fragments mapped (FPKM) was used to describe the transcription abundance of lncRNAs and mRNAs, which was quantified by cuffquant and cuffnorm. miRDeep2 was used to quantified the transcription abundance of miRNAs [37].

### Differentially expressed genes analysis

The differentially expressed lncRNAs (DELs) and differentially expressed mRNA (DEMs) were identified in stage I LUAD tissues compared with adjacent non-tumor tissues by Cuffdiff. *P*<0.05and |log_2_fold change|>1 was set as the cut-off of DELs and DEMs. Differentially expressed miRNA (DEMIs) in stage I LUAD tissues were filteredusing DEGseq package in R language. miRNAs with FDR<0.001 and |log_2_fold change|>1 were selected as DEMIs.

### DEL-DEM co-expression network construction

Pearson correlation coefficients (PCCs) indicating co-expression relationship between DELs and DEMs were calculated. DEML-DEM co-expression pairs with |PCC≥0.90| were retained for network construction [38]. The DEL-DEM co-expression network was visualized by Cytoscape (http://cytoscape.org/) [39].

### Prediction of target genes of DEMIs

miRWalk database (http://www.umm.uni-heidelberg.de/apps/zmf/mirwalk/), was used to predict the target-genes of DEMIs [40]. In our study, 6 algorithms including RNA22, miRanda, miRDB, miRWalk, PICTAR and Targetscan in miRWalk was used. Moreover, the genes, predicted by more than 4 out of 6 algorithms, were considered as the target-gene of DEMIs. The target-genes were overlapped with DEMs in stage I LUAD and DEMI-DEMs interaction pairs in a negative manner were subjected to construct network, and visualized by Cytoscape [39].

### DEL-DEMI-DEM network

First, The nearby protein-coding genes of DELs with distance<100kb were identified in GRCH38 reference genome. Those protein-coding genes of DELs were overlapped with DEMs and the nearby DEMs of DELs were identified. Then, the nearby DEMs of DELs were overlapped with those DEMs negatively targeted by DEMIs. Lastly, DEL-DEMI-DEM network was constructed and visualized by Cytoscape [39].

### Gene ontology and KEGG enrichment analysis

In order to understand the potential biological functions and signaling pathways of DELs in stage I LUAD, GeneCoDis3 (http://genecodis.cnb.csic.es/analysis) analysis were conducted to Gene Ontology (GO) biological process and Kyoto Encyclopedia of Genes and Genomes (KEGG) pathway enrichment [41]. Those Items with FDR<0.05 was filtered as significant enrichments.

### Quantitative real-time polymerase chain reaction (qRT-PCR)

Total RNA of TNM stage I LUAD tissues and adjacent non-tumor tissues were extracted by using Trizol (Invitrogen, Carlbad, CA, USA) according to the manufacture instructions. FastQuantcDNA and miRcute Plus (Tiangen, Beijing, China) and miRNA First-Strand cDNA Synthesis Kit (Tiangen, Beijing, China) was used to synthesize the cDNA of mRNA and miRNA, respectively. qRT-PCRreactions were performed by using SuperRealPreMix Plus SYBR Green Kit (Tiangen, Beijing, China) and miRcute Plus miRNAqPCR Detection Kit (Tiangen, Beijing, China) on Applied Biosystems 7500 (AppliedBiosystems, Foster City, CA, USA). GAPDH and U6 were used as internal control for mRNA and miRNA detection, respectively. The relative expression of candidate genes was calculated by using the 2^-ΔΔCT^ equation methods [42]. The PCR primers used in our study were shown in Supplementary Table 3. At least triple experiments were subjected to qRT-PCR verification.

### The expression levels of representative lncRNAs were verified in TANRIC database

The Cancer Genome Atlas (TCGA, https://tcga-data.nci.nih.gov/tcga/) is a public funded project, which produces data at the RNA level for various cancers including lung adenocarcinom. The Atlas of Noncoding RNAs in Cancer (TANRIC, http://ibl.mdanderson.org/tanric/_design/basic/index.html) is an open resource for interactive exploration of lncRNAs in the context of TCGA clinical and genomic data. In our study, TANRIC database was employed to retrieve the lncRNA expression profiling of LUAD from TCGA database. The difference of expression levels of representative lncRNAs between LUAD and adjacent non-tumor tissues were calculated, which were delineated by box-plot analysis.

### Receiver operating characteristic analyses

In order to assess the diagnostic value of candidate DELs in LUAD, receiver operating characteristic (ROC) analyses were performed using pROC package in R language. The area under the curve (AUC) under binomial exact confidence interval was calculated to generate the ROC curve.

### Statistical analysis

Mean ± standard deviation and independent-samples *t*-test was used in the statistical analysis. *P*<0.05 was considered as significant difference. * indicated *P*<0.05; ** indicated *P*<0.01 and *** indicated *P*<0.001.

## SUPPLEMENTARY MATERIALS FIGURES AND TABLES





## Abbreviations

LUAD: lung adenocarcinoma
lncRNA: long non-coding RNA
DEL: differentially expressed lncRNA
DEM: differentially expressed mRNA
DEMI: differentially expressed microRNA
KEGG: Kyoto Encyclopedia of Genes and Genomes
NSCLC: non-small cell lung cancer
EGFR-TKI: epidermal growth factor receptor tyrosine kinase inhibitor
ESCC: esophageal squamous cell carcinoma
GO: gene ontology
qRT-PCR: quantitative real-time polymerase chain reaction
ROC: receiver operating characteristic
AUC: area under the curve
PCC: Pearson correlation coefficient
TCGA: The Cancer Genome Atlas
TANRIC: The Atlas of Noncoding RNAs in Cancer.
