# Supplementary material for: Identification of dysregulated long non-coding RNAs/microRNAs/mRNAs in TNM I stage lung adenocarcinoma
Source: Oncotarget. 2017 Jun 16;8(31):51703–18. doi: 10.18632/oncotarget.18512 (PMC5584281; doi:10.18632/oncotarget.18512)
Supplement: Supplementary file 1 [file oncotarget-08-51703-s001.pdf]

Identification of dysregulated long non-coding RNAs/microRNAs/mRNAs in TNM I stage lung adenocarcinoma

SUPPLEMENTARY MATERIALS

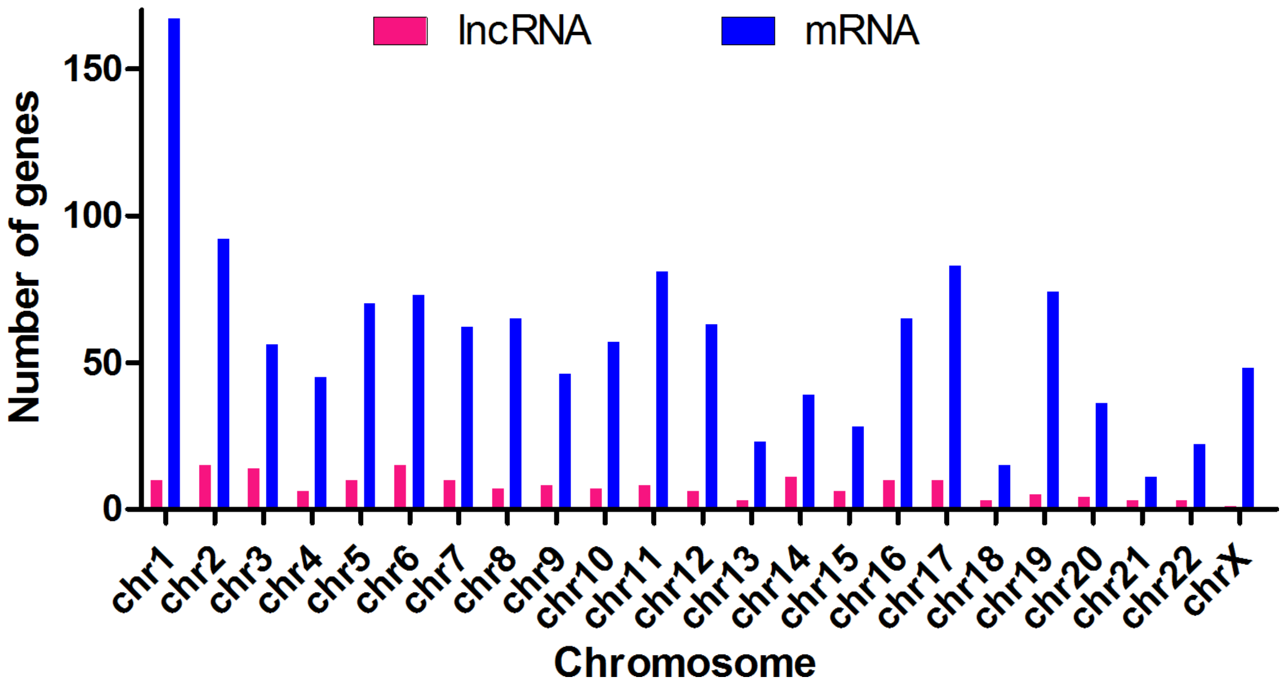

Supplementary Figure 1: The distribution of DEL and DEMs in stage I LUAD on chromosomes. Rose color and blue color indicated DELs and DEMs, respectively. The height of the bar represented the number of genes in each of chromosome. DELs were differentially expressed long non-coding RNAs and DEMs were differentially expressed protein-coding mRNAs.

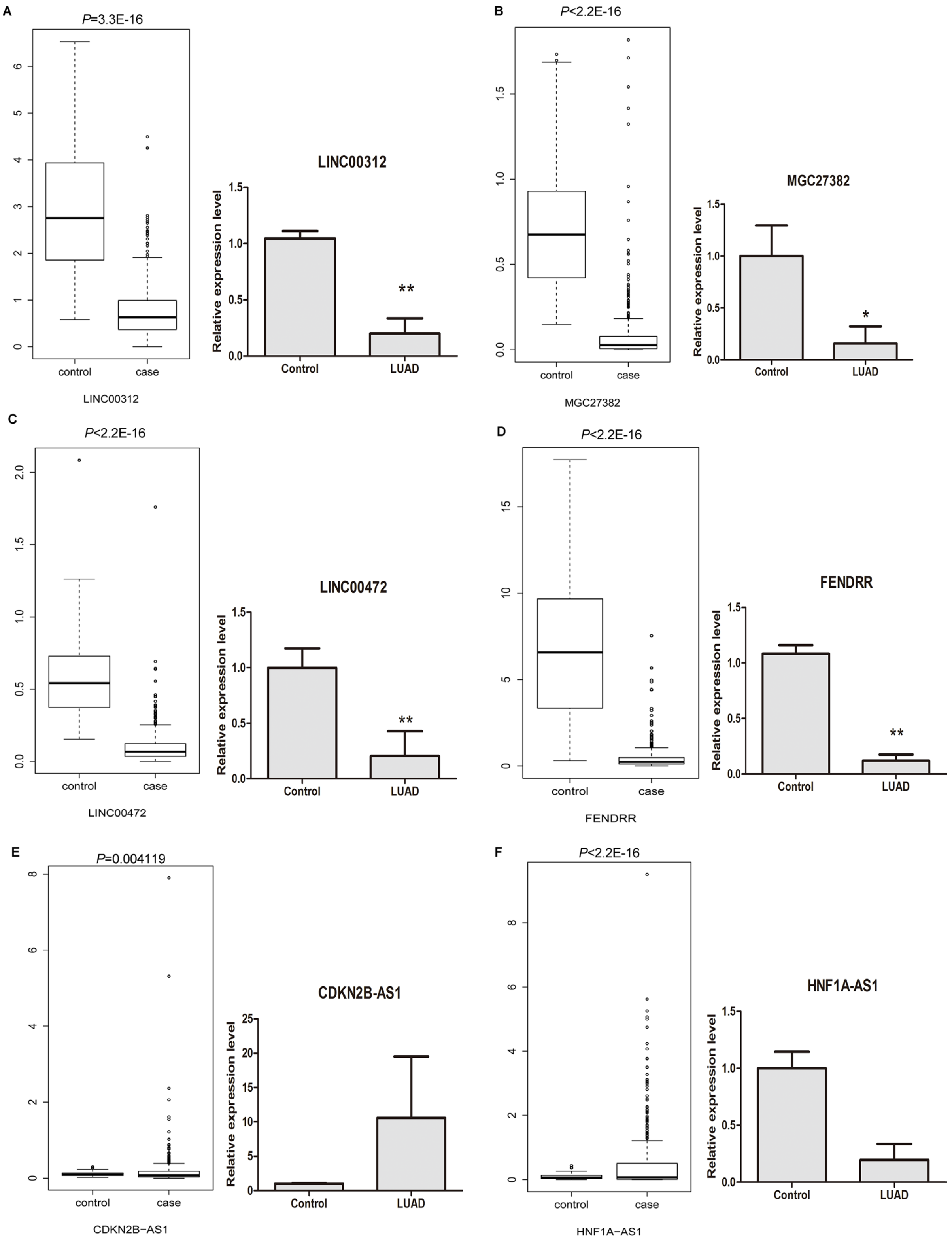

Supplementary Figure 2: The cross validation of the expression levels of candidate DELs in LUAD tissues based on TCGA database and qRT-PCR experiments. **(A)** LINC00312; **(B)** MGC27382; **(C)** LINC00472; **(D)** FENDRR; **(E)** CDKN2B-AS1; **(F)** HNF1A-AS1. Case group and control group indicated LUAD tissues and adjacent non-tumor tissues, respectivley. LUAD indicated stage I lung adenocarcinoma tissues. * represented *P*<0.05 and ** represented *P* <0.01.

Supplementary Table 1: Top 15 up- and down-regulated DEMIs in TNM I stage LUAD

miRNA	Log_2fold change	FDR
**UP-regulated (top 15)**		
hsa-miR-194-5p	4.919647924	0
hsa-miR-135b-5p	4.799147675	0
hsa-miR-135b-3p	4.605491061	1.51E-167
hsa-miR-215-5p	4.37125502	0
hsa-miR-192-5p	4.353530385	0
hsa-miR-192-3p	3.926256161	3.85E-196
hsa-miR-96-5p	3.717503264	0
hsa-miR-182-3p	3.619461169	7.87E-124
hsa-miR-146a-3p	3.451840115	1.41E-161
hsa-miR-182-5p	3.296540496	0
hsa-miR-183-5p	3.228662748	0
hsa-miR-200a-3p	3.012462133	0
hsa-miR-200a-5p	2.998877683	1.09E-269
hsa-miR-200b-5p	2.673829154	0
hsa-miR-200b-3p	2.499518611	0
**Down-regulated**		
hsa-miR-486-5p	-2.747918664	0
hsa-miR-338-3p	-2.295749558	0
hsa-miR-7641	-2.151187924	2.19E-121
hsa-miR-138-5p	-2.134098402	0
hsa-miR-451a	-2.042303381	0
hsa-miR-486-3p	-1.923790179	9.52E-81
hsa-miR-139-3p	-1.73410307	9.93E-133

DEMIs: differentially expressed miRNAs; LUAD: lung adenocarcinoma; FDR: false discovery rate.

Supplementary Table 2: The basic information of LUAD patients for RNA-sequencing

	Patient	Gender	Age	Subtype	TNM stage		Date of surgery	Sequencing/ qRT-PCR
1	1	female	63	LUAD	IA	T1aN0M0	2015/12/29	Sequencing
5	2	female	75	LUAD	IB	T2aN0M0	2016/1/13	Sequencing
9	3	male	52	LUAD	IA	T1aN0M0	2016/3/1	Sequencing
10	4	female	69	LUAD	IA	T1bN0M0	2016/3/1	Sequencing

LUAD: lung adenocarcinoma; TNM: tumor, node and metastasis; qRT-PCR: quantitative real-time polymerase chain reaction; subtype: the histological subtype of lung cancer.

Supplementary Table 3: Top 15 up- and down-regulated DEMIs in TNM I stage LUAD

See Supplementary File 1
