# Supplementary material for: Identification of dysregulated long non-coding RNAs/microRNAs/mRNAs in TNM I stage lung adenocarcinoma
Source: Oncotarget. 2017 Jun 16;8(31):51703–18. doi: 10.18632/oncotarget.18512 (PMC5584281; doi:10.18632/oncotarget.18512)
Supplement: Supplementary file 2 [file oncotarget-08-51703-s002.docx]

**Supplementary Table 2: The primers using in qRT-PCR**

| **Genes** | **Primers** |
| --- | --- |
| hsa-miR-200a-3p | Forward: UAACACUGUCUGGUAACGAUGU |
| hsa-miR-200b-3p | Forward: UAAUACUGCCUGGUAAUGAUGA |
| hsa-miR-200b-5p | Forward: CAUCUUACUGGGCAGCAUUGGA |
| hsa-miR-200c-5p | Forward: CGTCTTACCCAGCAGTGTTTGG |
| hsa-miR-338-3p | Forward: UCCAGCAUCAGUGAUUUUGUUG |
| hsa-miR-429 | Forward: UAAUACUGUCUGGUAAAACCGU |
| ADARB1 | Forward: GACCCTGTCTGTCAACACGG |
|  | Reverse: CCCACGTAAAAGGGAGGCTC |
| ADRB2 | Forward: CCTTCTACGTTCCCCTGGTGA |
|  | Reverse: GATGCCTAACGTCTTGAGGGC |
| ANKRD1 | Forward: AGTAGAGGAACTGGTCACTGG |
|  | Reverse: TGTTTCTCGCTTTTCCACTGTT |
| COL1A1 | Forward: GTGCGATGACGTGATCTGTGA |
|  | Reverse: CGGTGGTTTCTTGGTCGGT |
| MMP13 | Forward: TACACCTACACCGGCAAAAGC |
|  | Reverse: AAAACAGCTCCGCATCAACCT |
| MINCR | Forward:GTCTGTTTGGTGCCCTGGTC |
|  | Reverse:TGCGGTCTGAGGTCTCTAGC |
| LINC00963 | Forward:GAGTCCACGCCTGAACACTTC |
|  | Reverse:AGAATGTGGAAAGCAGCTGGG |
| NR2F2-AS1 | Forward:TCCAGATGAGGGAGCCAGTTG |
|  | Reverse:AAAGGTTAGCAGCTGAACCGC |
| LINC00515 | Forward:GAACGGAGCAGTGATGTGGTT |
|  | Reverse:GTGGAGGTCAGGACTACGGA |
| LBX2-AS1 | Forward:GCCTCCATACAGTTTGTCCCG |
|  | Reverse:TTGTCTGTATGCCATGCCAGG |
| LINC00162 | Forward:GGTGCCTCTTCCTCAGACATCT |
|  | Reverse:CAAGGAAAAGGACTGGGCTGG |
| LINC00312 | Forward: GGAGATGACGCTGTTGAA |
|  | Reverse: AGAGACCCAAAGGAATCAG |
| LINC00472 | Forward:AGCCAAACCATATCAGCTGCC |
|  | Reverse:CTGGAAGGTTGAGGCTGCAAT |
| FENDRR | Forward: AAACGCAACTACAGTGGACCC |
|  | Reverse: GGGCTTTTGACGAGTAGGCTG |
| CDKN2B-AS1 | Forward: CAACCTTGAACTCCCAGGCTC |
|  | Reverse: CCCAAATTCCTGCCACTTCCT |
| HNF1A-AS1 | Forward:TACCAACATGACGACCCCACT |
|  | Reverse:TGGCTAGAGAGTGGGGTTGAG |
| MGC27382 | Forward:GGCTTGCAAAAGTGGGGTTCT |
|  | Reverse:AAGTTTCCTGGCAAGCTACGG |
| GAPDH | Forward: GGAGCGAGATCCCTCCAAAAT |
|  | Reverse: GGCTGTTGTCATACTTCTCATGG |

qRT-PCR: quantitative real-time polymerase chain reaction.
